# Gut metabolite trimethylamine *N*-oxide induces aging-associated phenotype of midbrain organoids for the induced pluripotent stem cell-based modeling of late-onset disease

**DOI:** 10.3389/fnagi.2022.925227

**Published:** 2022-08-16

**Authors:** Youngsun Lee, Ji Su Kang, On-Ju Ham, Mi-Young Son, Mi-Ok Lee

**Affiliations:** ^1^Stem Cell Convergence Research Center, Korea Research Institute of Bioscience and Biotechnology (KRIBB), Daejeon, South Korea; ^2^Department of Bioscience, Korea University of Science and Technology (UST), Daejeon, South Korea

**Keywords:** brain organoid, midbrain, gut metabolite, TMAO, aging, neurodegenerative disease

## Abstract

Brain organoids are valuable research models for human development and disease since they mimic the various cell compositions and structures of the human brain; however, they have challenges in presenting aging phenotypes for degenerative diseases. This study analyzed the association between aging and the gut metabolite trimethylamine *N*-oxide (TMAO), which is highly found in the midbrain of elderly and Parkinson’s disease (PD) patients. TMAO treatment in midbrain organoid induced aging-associated molecular changes, including increased senescence marker expression (*P21, P16*), p53 accumulation, and epigenetic alterations. In addition, TMAO-treated midbrain organoids have shown parts of neurodegeneration phenotypes, including impaired brain-derived neurotrophic factor (BDNF) signaling, loss of dopaminergic neurons, astrocyte activation, and neuromelanin accumulation. Moreover, we found TMAO treatment-induced pathophysiological phosphorylation of α-synuclein protein at Ser-129 residues and Tau protein at Ser202/Thr205. These results suggest a role of TMAO in the aging and pathogenesis of the midbrain and provide insight into how intestinal dysfunction increases the risk of PD. Furthermore, this system can be utilized as a novel aging model for induced pluripotent stem cell (iPSC)-based modeling of late-onset diseases.

## Introduction

Aging is the degenerative alterations in the body with age, accompanied by physiological, behavioral, and psychological changes, and contributes to the onset of diseases. In particular, aging is closely related to neurodegeneration such as Parkinson’s disease (PD) in the central nervous system (CNS). Although various genetic and environmental factors that cause PD have been reported, aging is the greatest risk factor for PD. However, it remains unknown which specific mechanisms of aging induce the pre-parkinsonian state or what other mechanisms isolated from aging lead to PD (Pang et al., 2019). Understanding the detailed molecular mechanism of aging-related alteration and disease onset, especially in the human brain, will be a breakthrough in discovering treatments to cure.

Although numerous animal models are being studied to elucidate the relationship between aging and PD and identify the causes of diseases, these models face some challenges, including having different anatomical, and physiological characteristics from humans (Potashkin et al., 2010). As a method of studying human diseases by more closely mimicking the human microenvironment, induced pluripotent stem cell (iPSC) technology has recently been in the spotlight (Liu et al., 2018). In particular, brain organoids that can be produced through three-dimensional neuronal differentiation of human pluripotent stem cells (hPSCs) provide an advanced *in vitro* model for studying human brain development and disease by mimicking the cell types and tissue architecture of the human brain (Koo et al., 2019). However, since cellular reprogramming is known to erase aging-related features of patient cells, such as DNA damage, mitochondrial reactive oxygen species (ROS), nuclear envelope dysfunction, and epigenetic alteration (Simpson et al., 2021), it is limited to recapitulating the alterations of neurodegenerative diseases that show late-onset phenotypes with aging in induced pluripotent stem cell-based disease models. For instance, an increase in α-synuclein could be observed in an *in vitro* PD model using patient iPSC-derived midbrain organoids; however, Lewy body-like inclusions, a hallmark of PD, were not formed (Kim et al., 2019). This is why we need a model showing aging factors for PD study.

Given the importance of degenerative brain disease research, many studies are being conducted on methods to simulate them (Brunet, 2020; Slanzi et al., 2020). In iPSC-based studies, modeling for the implementation of an aging model including a progeria-induced model is being studied (Miller et al., 2013). However, the method through genetic modification is difficult to reproduce the phenomenon of natural aging (Azam et al., 2021). As a factor inducing such natural aging, the field of the gut microbiome is receiving attention recently (Ghosh et al., 2022). The gut microbiota plays an important role in various human health and diseases ranging from the immune system, metabolic disorders, and cancer, and shows potential as a biomarker (Zhu et al., 2020). Several studies have reported that gastrointestinal dysfunction is associated with PD risk (Fasano et al., 2015; Mukherjee et al., 2016; Schapira et al., 2017; Metta et al., 2022). Microbiota dysbiosis is related not only to atherosclerosis and stroke in the brain, but also to neurodegeneration such as Alzheimer’s disease (AD), autism, multiple sclerosis, and PD (Janeiro et al., 2018; Gandy et al., 2019; Glowacki and Martens, 2020). Based on the latest report on the formation of a unique microbiome with aging (Wilmanski et al., 2021), the aging-related microbiome may be involved in the pathogenesis of late-onset diseases, including PD.

Trimethylamine *N*-oxide (TMAO) is a metabolite produced by flavin monooxygenase 3 (FMO3) in the liver from trimethylamine (TMA), increases in human and mouse blood as well as cerebrospinal fluid (CSF) with age, and TMAO treatment induces an increase in the aging phenotype, neuronal degeneration, and cognitive impairment in SAMP8, 3X Tg-AD mice, and HUVECs (Ke et al., 2018; Li et al., 2018; Govindarajulu et al., 2020; Brunt et al., 2021). Although the detailed mechanism remains unknown, it has been reported that it induces endoplasmic reticulum (ER) stress, C/EBP homologous protein (CHOP), and ROS to pass through the blood-brain barrier as well as the peripheral organs, causing neurodegeneration and affecting the CNS (Govindarajulu et al., 2020; Rosario et al., 2020). Even though the role of TMAO in PD pathogenesis is largely unknown, Chen et al. (2020) reported that increased plasma levels of TMAO in PD patients are associated with PD severity and progression.

This study devised a method to induce age-related phenotypes in midbrain organoids for aging and PD modeling. The effects of TMAO treatment were analyzed in terms of midbrain aging and PD pathogenesis. Hence, this study proposes a new research model to study late-onset brain diseases in human organoids and provides insight into how the gut microbiota can make the brain old.

## Materials and methods

### Cell culture

H9 cells, a human embryonic stem cell line (WiCell, Madison, WI, United States) and human iPSCs (LRRK2^*G*2019*S*^ mutant PD-patient iPSC and gene-corrected iPSC) (Ha et al., 2020) were cultured in TeSR-E8 medium (STEMCELL Technologies, Vancouver, Canada) supplemented with 1% Pen/Strep (Gibco, Carlsbad, CA, United States) in matrigel (Corning, NY, United States) coated dishes. Cells were passaged using ReLeSR (STEMCELL Technologies, Vancouver, Canada), and the medium was changed on alternate days. This research with human embryonic stem cell and hiPSCs was approved by the Public Institutional Bioethics Committee designated by the Ministry and Welfare (MoHW) (Seoul, South Korea, IRB no. P01-201409-ES-01, P01-201802-31-001).

### Generation of midbrain organoid

Human pluripotent stem cells maintained in the TeSR-E8 medium were dissociated into single cells with Accutase (MERTK), and 1.0 × 10^4^ cells were seeded in an ultra-low attachment 96-well plate (S-bio, Hudson, NH, United States) for self-organization (Kwak et al., 2020). When the cell formed embryoid bodies, the media was replaced with EBM [DMEM/F12 (Gibco, Carlsbad, CA, United States) supplemented with 20% KSR (Gibco, Carlsbad, CA, United States), 50 μM Y27632 (Tocris, Bristol, United Kingdom), 3 μM CHIR99021 (Tocris), 1 μM IWP2 (Biogems, Westlake Village, CA, United States), 2 μM dorsomorphin (Sigma, St. Louis, MO, United States), 2 μM A83-01 (PeproTech, Rocky Hill, NJ, United States), 55 μM ß-mercaptoethanol (Gibco, Carlsbad, CA, United States), 3% FBS (Gibco, Carlsbad, CA, United States), 4 ng/ml bFGF (PeproTech), 1 μg/ml heparin (Sigma, St. Louis, MO, United States), 1% NEAA (Gibco, Carlsbad, CA, United States), 1% Pen/Strep, 1% GlutaMAX (Gibco, Carlsbad, CA, United States)], and after 24 h, it was replaced with BGM media (DMEM/F12: Neurobasal medium (1:1) supplemented with 1X N2 (Gibco, Carlsbad, CA, United States), 1X B27 w/o vitamin A (Gibco, Carlsbad, CA, United States), 3 μM CHIR99021, 1 μM IWP2, 2 μM dorsomorphin, 2 μM A83-01, 55 μM ß-mercaptoethanol, 1 μg/ml heparin, 1% NEAA, 1% Pen/Strep, and 1% GlutaMAX). After 2 days, FGF8 (PeproTech) and SAG (PeproTech) were added to the BGM media for mesencephalon patterning. The organoids were embedded in growth factor-reduced matrigel (Corning, NY, United States). Laminin (BD Science, Franklin Lakes, NJ, United States) and insulin (Thermo Scientific, Waltham, MA, United States) were added to the media, and CHIR99021, IWP2, dorsomorphin, and A83-01 were withdrawn. On day 9, the matrigel-embedded organoids were transferred to an ultra-low attachment 6-well plate and cultured on an orbital shaker at 60 rpm. The BMM medium [DMEM/F12: Neurobasal medium (1:1) supplemented with 1X N2, 1X B27 (Gibco, Carlsbad, CA, United States), 10 ng/ml BDNF (PeproTech), 10 ng/ml GDNF (PeproTech), 200 μM ascorbic acid (Sigma, St. Louis, MO, United States), 125 μM db-cAMP (Biogems), 55 μM ß-mercaptoethanol, 1 μg/ml heparin, 1% NEAA, 1% Pen/Strep, and 1% GlutaMAX] was changed on alternate days. After 4WM, 100 uM, or 1 mM of TMAO was continuously treated whenever the medium was changed once every 2 days.

### RNA isolation and qPCR

The organoids were washed with 1XPBS and lysed using easy-BLUE™. Total RNA extraction kit (iNtRON Biotechnology, Seongnam, Republic of Korea). RNA was isolated using chloroform and isopropanol. The RNA pellet was washed with cold 70% EtOH, and the concentration was measured using Nanodrop. All 1,200 ng RNA was synthesized as cDNA with Superscript IV Reverse Transcriptase (Thermo Scientific, Waltham, MA, United States). The amount of gene expression was analyzed using Fast SYBR™ Green PCR Master Mix (Applied Biosystems, Waltham, MA, United States) and primers for each marker. The expression level of each marker was normalized with *TBP*. The primer informations used in this study are provided in Supplementary Table 1.

### Immunocytochemistry

The organoids were fixed in 4% paraformaldehyde at room temperature for 6 h and washed with 1XPBS. Next, organoids were embedded in OCT (Sakura, Tokyo, Japan) for frozen blocks, and frozen organoids were cut into 7 μm slices using a cryostat (LEICA CM1520). Samples were blocked with 3% of BSA and 0.02% of sodium azide in 0.025% TBS-T for 1 h after permeabilization with 0.3% of tritonX-100 (Sigma, St. Louis, MO, United States) in 0.025% TBS-T for 1 h, and target protein was stained with 1:100 diluted primary antibodies against SOX2 (Seven Hills Bioreagent, Cincinnati, OH, United States, #WRAB1236), FOXA2 (Seven Hills Bioreagent, Cincinnati, OH, United States, #WRAB1200), LMX1a (Sigma, St. Louis, MO, United States, #ab10533), Tuj1 (BioLegend, San Diego, CA, United States, #802001), TH (Sigma, St. Louis, MO, United States, #T1299), MAP2 (R&D, Minneapolis, Minnesota, United States, #MAB933), glial fibrillary acidic protein (GFAP) (DAKO, #Z0334), pERK1/2 (Cell signaling, #9106s), pCREB (Cell signaling, #9198S), p53 (Santa cruz, biotechnology, Dallas, TX, United States, #sc-126), Lamin A/C (Santa cruz, biotechnology, Dallas, TX, United States, #sc-376248), CaMKII (Novus biologicals, #NB110-96869), BNDF (Alomone labs, Jerusalem, Israel, #ANT-010), Tri-Me-K9 (abcam, Cambridge, United Kingdom, #ab8898), PSD-95 (Invitrogen, Waltham, MA, United States, #MA1-046), Synaptophysin (abcam, Cambridge, United Kingdom, #ab32127), α-synuclein (Millipore, Billerica, MA, United States, #AB9850), pTau (Invitrogen, Waltham, MA, United States, MN1020), cleaved caspase 3 (Cell signaling, #9664s) at 4°C overnight. Slides were washed with 1X TBS-T and incubated with 1:400 diluted secondary antibodies (Alexa Fluor 488 goat anti-mouse IgG, Life Technologies, Alexa Fluor 555 donkey anti-rabbit, Life Technologies) with Hoechst (Thermo Scientific, Waltham, MA, United States) at room temperature for 1 h in deem light. The stained slides were observed under a fluorescence microscope (OLYMPUS, Shinjuku, Tokyo, Japan, U-TBI90) and a confocal microscope (ZEISS Oberkochen, Germany, LSM800).

### Enzyme-linked immunosorbent assay

According to the manufacturer’s instructions, we used a dopamine enzyme-linked immunosorbent assay (ELISA) kit to analyze the dopamine produced by midbrain organoids (Enzo, Basel, Swizerland, #ENZ-KIT188-0001). Briefly, conditioned media for each sample was added to each well in addition to the same volume of biotin detection antibody. The plate was set at 37°C for 45 min and washed three times. Next, the horseradish peroxidase (HRP) streptavidin conjugate working solution was added and set at 37°C for 30 min. The samples were then washed and incubated with the tetramethylbensidine (TMB) substrate for 15 min at 37°C in the deem light. The stop solution was immediately added, and the absorbance [optical density (OD)] was read using a microplate reader (SpectraMax, M3 Multi-Mode Microplate Reader) at 450 nm.

### Fontana-Masson staining (melanin stain)

We visualized melanin in midbrain organoids to confirm the accumulation of neuromelanin using Fontana-Masson (Abcam, Cambridge, United Kingdom, #ab150669). The samples were washed in distilled water and set in an ammonical silver solution at 60°C for 45 min. Next, it was washed with distilled water and set in 0.2% gold chloride solution for 30 s at room temperature. The samples were then washed and set in 5% sodium thiosulfate solution at room temperature for 2 min. Finally, nuclei and cytoplasm were stained with the nuclear fast red solution for 5 min. Before mounting, samples were dehydrated in 100% EtOH.

### Aggresome staining

The misfolded and aggregated protein were identified with the PROTEOSTAT Aggresome detection kit (Enzo, #ENZ-51035-K100). According to the manufacturer’s instructions, samples were washed three times with 1XPBS for 10 min each. And then incubated with dual detection reagents (Dilute Aggresome Detection Reagent 1:2000 and Hoechst 33342 1:1000 in 1X Assay Buffer) for 30 min at room temperature in deem light. After washing with 1XPBS, the samples were mounted and observed under a confocal microscope.

### Statistical analysis

All experiments were repeated three or more times and analyzed using Prism 6.0. Three to six organoid samples were analyzed for each group and used three sections per organoid. In each organoid section, three to five image was analyzed and image analysis proceeded with ImageJ software which quantified the fluorescence area of immunostaining in the same fluorescence intensity states and normalized with the fluorescence area of Hoechst. The non-parametric (Mann–Whitney) test was used for statistical analyses. Data values are presented as AVE ± SD. Statistical significance was set at *P* < 0.05, *P* < 0.01, *P* < 0.001, *P* < 0.0001 (*, ^**^, ^***^, ^****^).

## Results

### Generation of midbrain organoid

**FIGURE 1:**
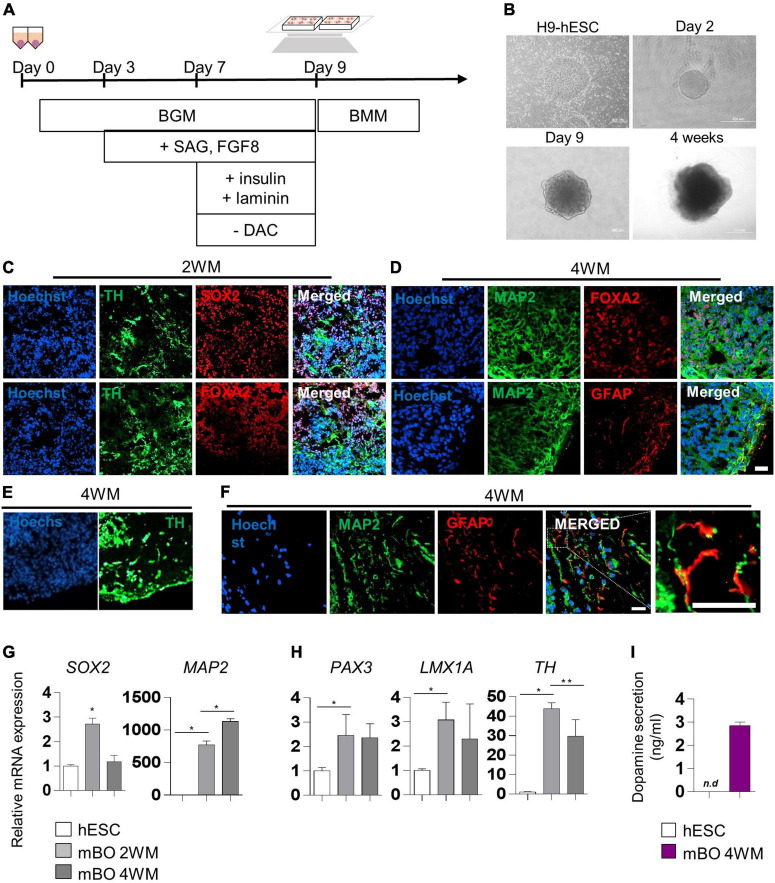
Characterization of midbrain organoids. **(A)** Schematic method of the midbrain organoids generation. **(B)** Representative phase-contrast images of human embryonic stem cell (hESC) and midbrain organoids at day 2, day 9, and 4 weeks of maturation. **(C–E)** Representative Immunostaining fluorescence (IF) Images showing the expression of the differentiation markers (*SOX2, FOXA2, TH, MAP2, and GFAP*) in midbrain organoids after 2, 4 weeks of maturation. Hoechst was used for counter nuclei staining. **(F)** High-resolution images of MAP2 and GFAP in midbrain organoids. **(G,H)** The relative mRNA expression level of differentiation markers (*SOX2, PAX3, LMX1a, TH, and MAP2*) of undifferentiated hESC and midbrain organoids (2WM, 4WM). **(I)** Quantification of dopamine secretion to conditioned media from undifferentiated hESC and midbrain organoids (4WMs). Data are AVE ± SD [*P* < 0.05(*), *P* < 0.01(**)]. (Scale bar = 20 μm).

We generated midbrain organoids from human embryonic stem cells (hESCs) to mimic the identity of the human midbrain, as previously reported by Kwak et al. (2020). Differentiation into midbrain organoids was performed by a guided neural-differentiation protocol (Figures 1A,B) and characterized by immunostaining for neuronal-specific markers (Figures 1C,D). At 2 weeks after maturation (2WM), we observed the expression of the neuronal stem cell marker SRY-box transcription factor 2 (SOX2), as well as the dopaminergic progenitor marker forkhead box protein A2 (FOXA2), which indicated midbrain-like specification of organoids. In addition, the expression of the dopaminergic neuronal marker tyrosine hydroxylase (TH) began to be detected in 2WM midbrain organoids (Figure 1D). In 4WM, the astrocyte marker GFAP was detected in organoids with microtubule-associated protein 2 (MAP2), TH and FOXA2, indicating glial differentiation in midbrain organoids (Figures 1D,E). In the magnified image, it was confirmed that GFAP-positive astrocytes were surround MAP2-positive neurons (Figure 1F).

The relative mRNA expression of cell type-specific genes was analyzed using RT-PCR for a quantitative comparison. The neural stem cell marker *SOX2* increased in 2WM organoids and then decreased in 4WM organoids, and the expression of the mature neuronal marker *MAP2* gradually increased with differentiation (Figure 1G). Dopaminergic neuronal markers (*PAX3*, *LMX1a*, and *TH*) maintained high at 4 weeks, but decreased slightly compared to 2 weeks. This is presumed to be due to the change in the relative portion of dopaminergic neurons in midbrain organoids with the increase of astrocytes and non-dopaminergic neurons (Figure 1H). Furthermore, the dopamine ELISA assay (Figure 1I) showed increased dopamine secretion (about 3 ng/ml) in the conditioned medium of single midbrain organoid cultures, demonstrating functional maturation of dopaminergic neurons in 4WM organoids.

### Increase of cellular stress and aging features by trimethylamine *N*-oxide treatment

**FIGURE 2:**
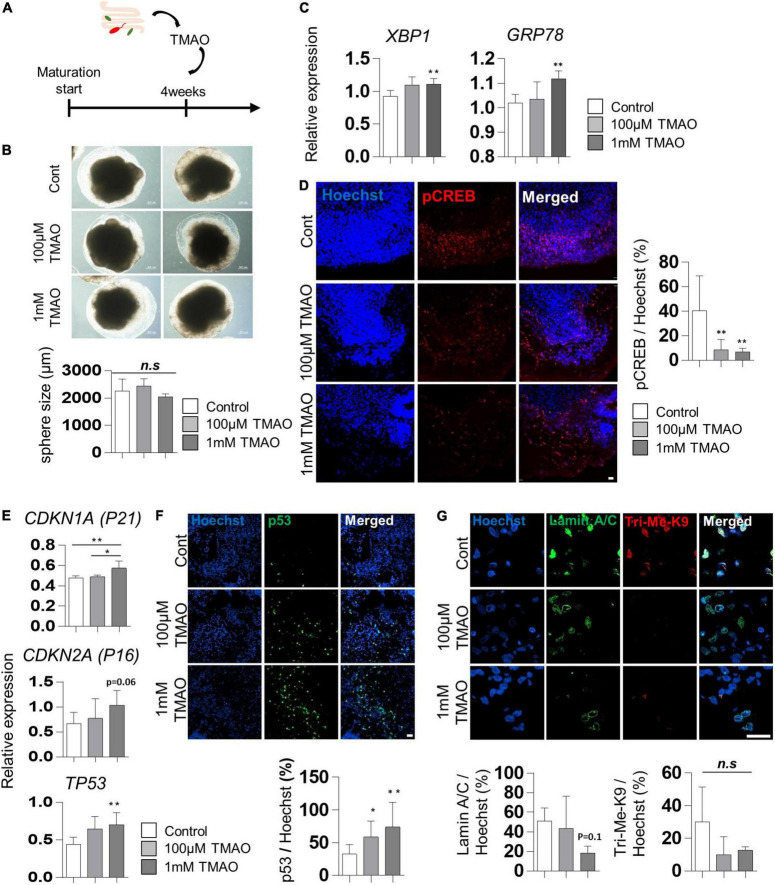
Aging-associated molecular phenotypes of trimethylamine *N*-oxide (TMAO)-treated midbrain organoids. **(A)** Schematics of TMAO treatment in midbrain organoid. **(B)** Representative phase-contrast images (upper) and quantification of organoid size (lower). **(C)** Relative mRNA expression levels of *XBP1* and *GRP78*. **(D)** Relative IF images (left) and quantification of phosphorylated cAMP response element-binding protein-positive cells in indicated groups (8WMs). Hoechst was used for counter nuclei staining. **(E)** The relative mRNA expression level of *CDKN1A, CDKN2A, and TP53*. **(F)** Representative IF images (upper) and quantification (lower) of the p53-expressing cells in the indicated organoid group. **(G)** Representative IF images (upper) and quantification (lower) of the Lamin A/C and Tri-Me-K9 in the indicated organoid group. Data are AVE ± SD [*P* < 0.05(*), *P* < 0.01(**)].

To evaluate the effects of TMAO on the neuropathological differentiation of the midbrain, we started treating 4WM organoids with TMAO at two concentrations (100 μM and 1 mM) for 26 weeks and analyzed the pathophysiological changes in organoids (Figure 2A). After 4 weeks of TMAO treatment (8WMs organoids), there was no significant difference in the diameter of the spheres and apoptotic cells between the control and TMAO treatment groups (Figure 2B and Supplementary Figures 1A,B).

Previous studies have reported that TMAO induces ER stress in the aged population and PD patients (Govindarajulu et al., 2020). In our experiments, the expression of the ER stress-related genes, X-box binding protein 1 (*XBP1*) and glucose regulatory protein 78 (*GRP78*), was slightly increased in TMAO-treated organoids (Figure 2C), similar to that reported in pancreatic acinar cells (Yang and Zhang, 2021). In addition, phosphorylation of cAMP response element-binding protein (CREB), known to be inhibited by ER stress-mediated PERK activation (Kikuchi et al., 2016), was examined by immunostaining. The results showed a significant decrease of phosphorylated CREB (pCREB) by TMAO treatment in midbrain organoids (Figure 2D), implying the activation of ER stress by TMAO in midbrain organoids.

We analyzed changes in well-known senescence-associated aging markers in midbrain organoids to determine whether TMAO could induce aging-associated alterations in midbrain organoids. The expressions of *CDKN1A (P21), CDKN2A (P16)*, and *TP53* were analyzed by qPCR in 8WM organoids with or without treatment with TMAO for 4 weeks. The *TP53* expression was increased in organoids treated with TMAO at 100 μM and 1 mM concentrations compared to the control (Figure 2E). The expression of *CDKN1A* and *CDKN2A* also increased at the 1 mM TMAO treatment group (Figure 2E). In addition, immunostaining showed nuclear accumulation of p53 in TMAO-treated organoids (Figure 2F).

Moreover, we observed differences in the immunostaining results for lamin A/C and Tri-Me-K9, which are epigenetic aging markers reported to maintain the stability of the nuclear architecture and structure of chromatin (Scaffidi and Misteli, 2006). These proteins are defective with aging and lamin A/C and Tri-Me-K9 alteration, leading to telomere instability (Burla et al., 2016). Lamin A/C was decreased in the 1 mM TMAO treatment group compared to the control group. Tir-Me-K9 tended to decrease with TMAO (Figure 2G). These results demonstrate that TMAO reduces repressive epigenetic aging markers and decreases the structural stability of the nucleus and chromatin in midbrain organoids.

### Weakened dopaminergic neuron protection by trimethylamine *N*-oxide treatment

**FIGURE 3:**
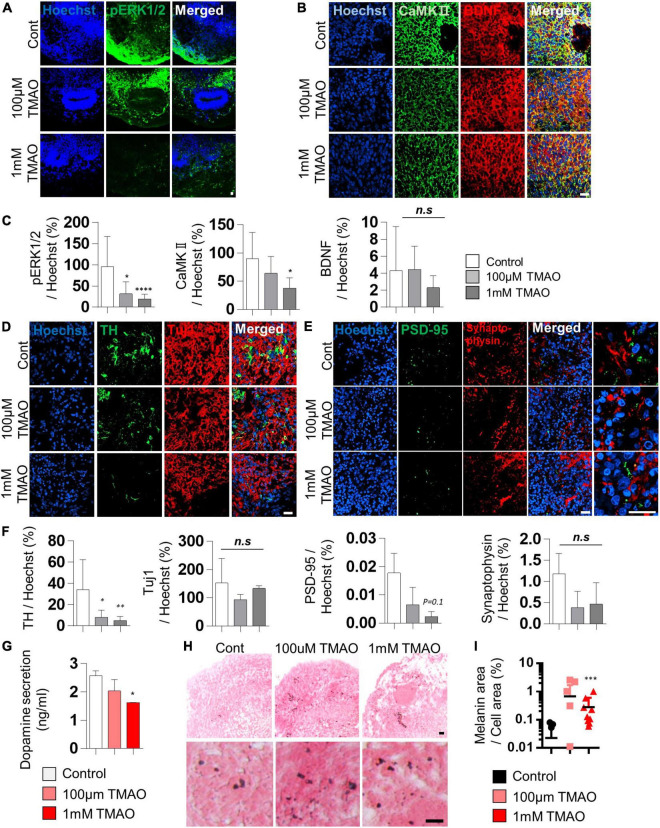
Neurodegeneration phenotypes of trimethylamine *N*-oxide (TMAO)-treated midbrain organoids. **(A,B)** Representative IF images showing the ERK phosphorylation **(A)** and CaMKII/BDNF **(B)** in the indicated organoid group. Hoechst was used for counter nuclei staining. **(C)** Quantifications of the ERK phosphorylation (left), CaMKII (middle), and BDNF (right) in the indicated organoid group. **(D,E)** Representative IF images showing the TH- and TUJ-1 positive neurons **(D)** and synaptic marker expression (PSD-95 and Synaptophysin) **(E)** in the indicated organoid group. Hoechst was used for counter nuclei staining. **(F)** Quantifications of the TH, TUJ-1 (left), PSD-95, and Synaptophysin (right) in the indicated organoid group. **(G)** Quantification of dopamine secretion to conditioned media from indicated organoids. **(H)** Representative Fontana-Masson staining images for neuromelanin at 8 weeks of maturation in midbrain organoids. **(I)** Quantifications of Fontana-Masson staining. Data are AVE ± SD [*P* < 0.05(*), *P* < 0.01(**), *P* < 0.001(***)]. (Scale bar = 20 μm).

Brain-derived neurotrophic factor regulates TH expression. It plays a protective role in dopaminergic neurons through CREB phosphorylation triggered primarily by MAPK/ERK1/2 and calmodulin-dependent protein kinase II (CaMKII) (Palasz et al., 2020). The decrease in phosphorylation of CREB by TMAO treatment was confirmed in Figure 2D. We further tested the intracellular signaling changes of BDNF supplemented in culture media to maintain the midbrain organoids. The phosphorylation of ERK1/2 in differentiated neuronal regions was dramatically reduced (Figures 3A,C), and CaMKII was decreased in TMAO-treated organoids. In contrast, BDNF expression was not significantly different (Figures 3B,C), implying impaired BDNF signaling.

Next, we compared the expression of TH, a marker of dopaminergic neurons, in midbrain organoids 4 weeks after TMAO treatment. Immunostaining showed that the number of TH-positive dopaminergic neurons was decreased in TMAO-treated organoids (Figures 3D,F). However, there was no significant difference in Tuj1, indicating that dopaminergic neurons were more susceptible to TMAO treatment than other neurons. The decrease in dopamine secretion from organoids was concentration-dependent following TMAO treatment (Figure 3G).

Synaptophysin and post-synaptic density protein 95 (PSD-95), pre- and post-synaptic proteins, respectively, were stained and quantified to confirm changes in synaptic proteins. PSD95 was decreased by TMAO treatment and Synaptophysin levels also slightly decrease with TMAO treatment, although this was insignificant (Figures 3E,F and Supplementary Figure 2), suggesting TMAO may affect synaptic dysfunction.

Furthermore, we detected neuromelanin in organoids synthesized from L-dopa and reportedly accumulates in the aged midbrain. Neuromelanin was produced in 8WM organoids, and its accumulation was significantly increased in the 1 mM TMAO treatment group than in the control group (Figures 3H,I). Altogether, the results show that TMAO-treated midbrain organoids could mimic parts of the cellular features of the aged midbrain, including loss of dopaminergic neurons, functional decline, and neuromelanin accumulation.

### Astrocyte activation by trimethylamine *N*-oxide treatment

**FIGURE 4:**
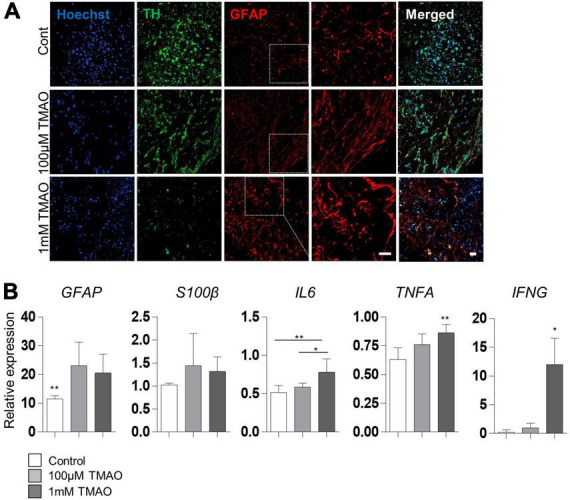
Astrocyte activations in trimethylamine *N*-oxide (TMAO)-treated midbrain organoids. **(A)** Representative IF images showing the TH-positive dopaminergic neurons and glial fibrillary acidic protein (GFAP)-positive astrocytes in indicated organoids. Hoechst was used for counter nuclei staining. **(B)** Relative mRNA expression level of GFAP and mRNA expression level of activation astrocytic markers, *GFAP, S100β, IL6, TNFA, and IFNA* in indicated organoids (8WMs). Data are AVE ± SD [*P* < 0.05(*), *P* < 0.01(**)]. (Scale bar = 20 μm).

Given that astrocyte activation is one of the features of the aged brain, we confirmed the expression of the astrocyte activation marker, GFAP, in TMAO-treated midbrain organoids for 26 weeks. GFAP expression was enhanced by TMAO treatment, and astrocytes showed more activating morphology in the 1 mM TMAO treatment group, while TH-positive neurons were reduced (Figure 4A).

For quantitative analysis, *GFAP and hS100β* expression was examined by RT-PCR in 8WM organoids. As expected, *GFAP* expression was significantly increased in the TMAO treatment groups than in the control group. In addition, the expression of inflammatory cytokines, interleukin 6 (*IL6*) and interferon-γ (*IFNG*) was increased in the 1 mM treatment group compared to that in the control group. Still, there was no significant difference between the TMAO 100 μM treatment group and the control. Tumor necrosis factor-α (*TNFA*) levels were significantly increased by TMAO treatment (Figure 4B). These data show that TMAO might induce astrocyte-mediated inflammatory responses in midbrain organoids.

### Increase of abnormal protein aggregation by trimethylamine *N*-oxide treatment

**FIGURE 5:**
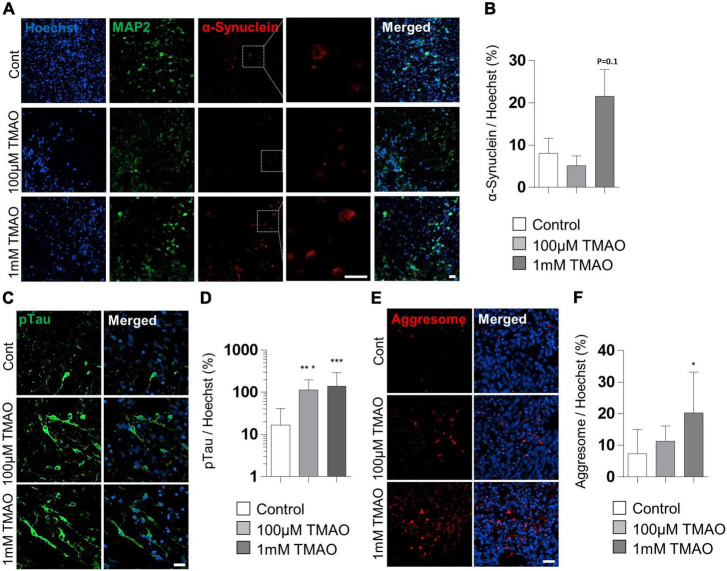
Parkinson’s disease (PD)-associated phenotypes in trimethylamine *N*-oxide (TMAO)-treated midbrain organoids. **(A)** Representative IF images showing the microtubule-associated protein 2 (MAP2) and phosphorylated α-synuclein (ser129) in the indicated organoids (30WMs). **(B)** Quantification of phosphorylated α-synuclein (ser129) accumulation in neurons. **(C)** Representative IF images showing the phosphorylated-Tau (ser202/thr205) in the indicated organoids (30WMs). **(D)** Quantification of pTau accumulation in indicated organoids. **(E)** Representative IF images showing the aggresome in the indicated organoids (30WMs). **(F)** Quantification of aggresome in indicated organoids. Data are AVE ± SD [*P* < 0.05(*), *P* < 0.01(**), *P* < 0.001(***)]. (Scale bar = 20 μm).

To verify the association between TMAO and PD pathogenesis, we confirmed the phosphorylation of the α-synuclein protein at Ser-129 residues, a representative pathophysiological modification in PD (Anderson et al., 2006). Immunostaining of the TMAO-treated midbrain organoids for 26 weeks revealed reactive signals in the intracellular space of neurons (Figure 5A). Compared to the control group, the 1 mM TMAO treatment group showed an increase in phosphorylation at Ser-129, although not in 100 μM TMAO-treated organoids (Figures 5A,B).

Furthermore, phosphorylation of Tau at Ser202/Thr205 was increased by TMAO treatment. In the 1 mM TMAO treatment group, Tau accumulation was observed in the soma and the axons (Figures 5C,D). After confirming the increase in phosphorated α-synuclein protein and Tau, protein aggregation was confirmed by aggresome staining to determine whether abnormal aggregation of the protein increased. As a result, aggresome was significantly increased in 1 mM TMAO-treated organoids compared with a control group (Figures 5E,F).

## Discussion

Since more than 90% of patients with neurodegenerative disorders, such as PD and AD, are sporadic cases, it is important to understand aging-associated alterations in the human brain and identify the relationship between age-related neurodegeneration and pathogenesis (Bekris et al., 2010; Slanzi et al., 2020). Herein, we investigated PD-associated predispositions of midbrain organoids by the microbiome metabolite TMAO, which increases with age. This study not only elucidates the role of TMAO in midbrain aging but also proposes a novel alternative method for studying late-onset degenerative brain disorders in hPSC-derived organoids.

Although an increase of TMAO has been reported in patients with PD, its role in midbrain aging and PD pathogenesis is largely unknown (Chen et al., 2020). In midbrain organoid models, we found that TMAO treatment can induce several features of the aged human brain, including increased cellular senescence and epigenetic aging markers, impaired neuronal function, glial cell activation, and inflammation (Mattson and Arumugam, 2018). Furthermore, we found that TMAO leads to phenotypes similar to the representative phenotype of PD, including loss of dopaminergic cells, increased phosphorylated alpha-synuclein, and phosphorylation of Tau. These results suggest that TMAO may play a crucial role in not only aging but also Parkinson’s pathogenesis in the midbrain.

Increased senescent cells are a hallmark of aging, and the expression of p21 and p16 is closely related to cellular senescence (Bernardes de Jesus and Blasco, 2012; Capparelli et al., 2012; Lopez-Otin et al., 2013). In our organoid model, we observed an increased expression of p53, p21, and p16 following treatment with TMAO. In addition, epigenetics, known as an accurate cellular clock and epigenetic change, are strong indicators of biological aging (Galow and Peleg, 2022). Long-term TMAO treatment resulted in a tendency to decrease H3K9me3 in midbrain organoids, suggesting the possibility of epigenetic aging. Furthermore, we found defective nuclear lamina in the TMAO-treated organoids. As the a-type lamina (lamin A/C) assists in maintaining the nuclear structure and chromatin throughout the nucleus, it is associated with telomere distribution and function maintenance (Scaffidi and Misteli, 2006; Burla et al., 2016). These results suggest that the gut metabolite TMAO could induce cellular senescence-like molecular changes in midbrain organoids. Given that senescence cells accelerate tissue aging (Xu et al., 2018), TMAO-induced cellular senescence may play a role in midbrain organoid aging.

The decline in the number and function of dopaminergic neurons with normal aging is well-known, and excessive loss of dopaminergic neurons induces PD (Noda et al., 2020). We found that TMAO could induce organoid changes similar to the dysfunction of the aged midbrain. TMAO-treated organoids showed a progressive loss of dopaminergic neurons and reduced dopamine production. Moreover, decreased expression of PSD-95 in TMAO-treated organoids may imply synaptic dysfunction. On the other hand, the expression of the astrocyte marker GFAP was increased by TMAO. Increased GFAP expression is a common feature of reactive/activated astrocytes in the aged brain (Palmer and Ousman, 2018). In addition, we found increased levels of inflammatory cytokines, IL-6, TNFα, and IFNγ, in TMAO-treated organoids. Inflammation plays an important role in neurodegeneration and induction of aging-related mechanisms (Calabrese et al., 2018). The gut microbiome has also attracted attention as one of the factors involved in inflammaging (Franceschi et al., 2018). According to our results, TMAO may play a role in astrocyte-mediated inflammation response. However, since neurons, as well as glial cells, can secrete the pro-inflammatory cytokine IL6 (Sun et al., 2017), it is still possible that the increase in inflammatory cytokines by TMAO was caused by neurons, not activated astrocytes.

Moreover, we observed increased production of neuromelanin in TMAO-treated midbrain organoids. Neuromelanin synthesized from L-dopa is only found in primates, especially to be high in humans found in the substantia nigra and locus coeruleus (Fedorow et al., 2005). Considering the accumulation of neuromelanin in the elderly, it is presumed to be related to aging (Zucca et al., 2017). The increased accumulation of neuromelanin has been reported in patients with PD. Still, its role has rarely been reported, and it is also difficult to study due to the absence of neuromelanin in experimental models. Therefore, a TMAO-treated midbrain organoid model may be useful to study the role of neuromelanin in brain aging and PD pathogenesis.

Typical histological phenotypes of PD, such as Lewy bodies, were not observed in the TMAO-treated organoids. However, some predispositions to PD have been identified. As mentioned above, a decrease in the number and function of dopaminergic neurons was confirmed, and the phosphorylation of α-synuclein (Ser-129) was also increased in TMAO-treated organoids. In addition, an increase in pTau and aggresome was confirmed in our generated midbrain organoids. Although pTau has not been considered a pathophysiological feature of PD, it has recently been reported that pTau is increased in 50% of PD patients (Zhang et al., 2018). In particular, it is associated with tauopathy in sporadic PD. Also, an increase in pTau aggregation is a representative pathophysiological feature of AD. Therefore, the results of our study demonstrate the possibility of sporadic neurodegenerative disease modeling related to aging.

Recently, it was reported that TMAO induces CREB dephosphorylation *via* ER stress-mediated PERK phosphorylation, which leads to deficits in synaptic plasticity in an Alzheimer’s mouse model (Chen et al., 2019; Govindarajulu et al., 2020; Yang and Zhang, 2021). In our midbrain organoid models, we also found a significant decrease in the pCREB levels in the TMAO treatment groups. In addition, the ER stress markers, *XBP1*, and *GRP78*, were increased by TMAO treatment. ER stress may play an important role in the TMAO-mediated midbrain organoid aging-like phenotype. Additionally, we newly demonstrated significant dephosphorylation of ERK and reduction of CaMKII, which is known to play an important role in neuroprotection in a BDNF-dependent manner (Palasz et al., 2020). These results suggest that TMAO may have an important inhibitory effect on BDNF signaling, which can induce neurodegeneration in midbrain organoids. Further studies on the precise mechanisms and epidemiology of this condition are needed.

Although brain organoids provide a research model similar to the developing human brain, the aging signature remains a major obstacle to degenerative disease research. Currently, the most widely used method of inducing aging in organoids is long-term culture, but there are issues such as an increase in the study period, cost, labor and risk of contamination, and it also causes considerable damage in healthy-donor organoids, such as a massive increase in apoptosis. In this study, we suggest a novel method to accelerate the aging signature in stem cell organoids, especially midbrain organoid aging. We tried to recapitulate the natural aging in midbrain organoids by mimicking the increased gut metabolite TMAO in the elderly and diseased individuals. This method has the advantage of being able to simply induce the aging phenotypes without genetic modification, and it can recapitulate the primate-specific feature of midbrain aging such as the increase of neuromelanin. Thus, it will contribute to understanding the degenerative changes and pathogenesis in the human midbrain.

The application of TMAO-induced aging models in patient-derived iPSCs could lead to the advance of degenerative disease models. In our experiments, we generate midbrain organoids from a PD patient-derived iPSCs harboring LRRK2^*G*2019*S*^ mutation and isogenic WT iPSCs by gene correction (Supplementary Figure 3A). Unfortunately, because of the difference in the initial size growth and the degree of differentiation between mutant cells and correction cells, we could not thoroughly analyze the effect of TMAO treatment on PD pathogenesis (Supplementary Figures 3B–F). However, it was confirmed that TMAO treatment in these iPSC-derived organoids induced aging-related alteration of midbrain organoids, including reduced TH+ cells, decreased dopamine secretion, increased expression of p53 and p21, and decreased pERK (Supplementary Figures 4A–F). Our preliminary analysis with PD-patients iPSC has not shown an additional role of TMAO in PD pathogenesis, but the detailed relevance of PD and TMAO should be elucidated using more patient-based midbrain organoids. It is very interesting to see whether typical disease phenotypes such as Lewy body formation, which were difficult to reproduce in existing models, can be reproduced in TMAO-induced aging models.

It is also possible that TMAO is involved in midbrain aging but does not play an important role in PD pathogenesis. Considering that the role of TMAO in AD has been reported in mouse experimental animal models, it will be interesting to compare specific responses in various neuronal regions using regional specific organoids. By analyzing the TMAO response for each cell level, it is also possible to study the interaction between each cell type and the relationship between cellular senescence, neurodegeneration, and late-onset of disease. The absence of cell types such as blood vessels and microglia in current midbrain organoids will still act as a limitation in disease models using TMAO-induced aging.

In conclusion, we developed a TMAO-treated midbrain organoid model as a novel method to study midbrain aging in humans. The aging-like phenotypes of midbrain organoids were confirmed by increased expression of senescence markers, decreased expression tendency of repressive histone markers, neural degeneration, and neuromelanin accumulation. Although the TMAO-treated midbrain organoid model has limitations in reflecting all complex factors of natural aging, it will be useful for studying the mechanisms of brain challenges in the aged gut environment. Moreover, the combination of TMAO-induced aging organoids with genetic PD modeling, such as the LRRK2 mutation, would provide an opportunity to study the role of TMAO in the pathogenesis of PD, which could be an advanced human PD model.

## Data availability statement

The original contributions presented in this study are included in the article/Supplementary material, further inquiries can be directed to the corresponding authors.

## Ethics statement

This research with human embryonic stem cells and hiPSCs was approved by the Public Institutional Bioethics Committee designated by the Ministry and Welfare (MoHW) (Seoul, South Korea, IRB nos. P01-201409-ES-01, P01-201802-31-001).

## Author contributions

YL, M-YS, and M-OL: conceptualization, manuscript writing, and review. YL, JK, and O-JH: validation and formal analysis. All authors have read and agreed with the published version of the manuscript.

## Abbreviations

TMAO: trimethylamine *N*-oxide
PD: Parkinson’s disease
AD: Alzheimer’s disease
hPSCs: human pluripotent stem cells
ROS: reactive oxygen species
FMO3: flavin monooxygenase 3
TMA: trimethylamine
ER: endoplasmic Reticulum
CHOP: C/EBP homologous protein
CNS: central nervous system
hESC: human embryonic stem cell
CSF: cerebrospinal fluid
OD: optical density
SOX2: SRY-box transcription factor 2
FOXA2: forkhead box protein A2
TH: tyrosine hydroxylase
GFAP: glial fibrillary acidic protein
MAP2: microtubule-associated protein 2
*XBP1*: X-box binding protein 1
*GRP78*: glucose regulatory protein 78
CREB: cAMP response element-binding protein
pCREB: phosphorylated CREB
BDNF: brain-derived neurotrophic factor
CaMKII: calmodulin-dependent protein kinase II
PSD-95: post-synaptic density protein 95
*IL6*: interleukin 6
*IFNG*: interferon-γ
*TNFA*: tumor necrosis factor-α
RT-PCR: real-time PCR
HRP: horseradish peroxidase
TMB: tetramethylbensidine.
