# Supplementary material for: Gut metabolite trimethylamine N-oxide induces aging-associated phenotype of midbrain organoids for the induced pluripotent stem cell-based modeling of late-onset disease
Source: Front Aging Neurosci. 2022 Aug 16;14:925227. doi: 10.3389/fnagi.2022.925227 (PMC9426463; doi:10.3389/fnagi.2022.925227)
Supplement: Supplementary file 1 [file Data_Sheet_1.docx]

Supplementary Material

**Table S1 | List of primers used in this study**

| Quantitative PCR | |  |
| --- | --- | --- |
| **Gene** | **Forward primer** | **Reverse primer** |
| *SOX2* | AGAACCCCAAGATGCACAAC | ATGTAGGTCTGCGAGCTGGT |
| *PAX3* | TTTCCGTTTCGCCTTCACCT | ACGATCTTGTGGCGGATGTG |
| *LMX1A* | AAAGCGCGATCGACACCTC | TCCCGGTAGAAGCAGGTGGT |
| *TH* | GGGCGTTGTAAGCAGAACG | AAGGCCCGAATCTCAGGCT |
| *MAP2* | CTCTGCAACAAACCAGTGGC | CTCTGAGGGTGGTGCTTCTG |
| *XBP1* | CCCTCCAGAACATCTCCCCAT | ACATGACTGGGTCCAAGTTGT |
| *GRP78* | CATCACGCCGTCCTATGTCG | CGTCAAAGACCGTGTTCTCG |
| *CDKN1A* | GCCATTAGCGCATCACAGT | CTGGGGATGTCCGTCAGAAC |
| *CDKN2A* | TGTGCTGGAAAATGAATGCTCTG | GTCCCTCAGGTGAGGACTGA |
| *TP53* | AAAGCTGTTCCGTCCCAGTA | GGACCTCCTAACCTGTGGCT |
| *GFAP* | GAGAACCGGATCACCATTCC | CCCAGTCTGGAGCAACCTAC |
| *IL6* | GGCACTGGCAGAAAACAACC | GCAAGTCTCCTCATTGAATCC |
| *TNFA* | TGGAGAGTGAACCAGGACCA | AATTCGCATAACTGCGTGGC |
| *IFNG* | TCCGCTACATCTGAATGACCTGCA | TTGGCTTTTCAGCTCTGCATCGTT |
| *S100β* | TGGCCCTCATCGACGTTTTC | ATGTTCAAAGAACTCGTGGCA |


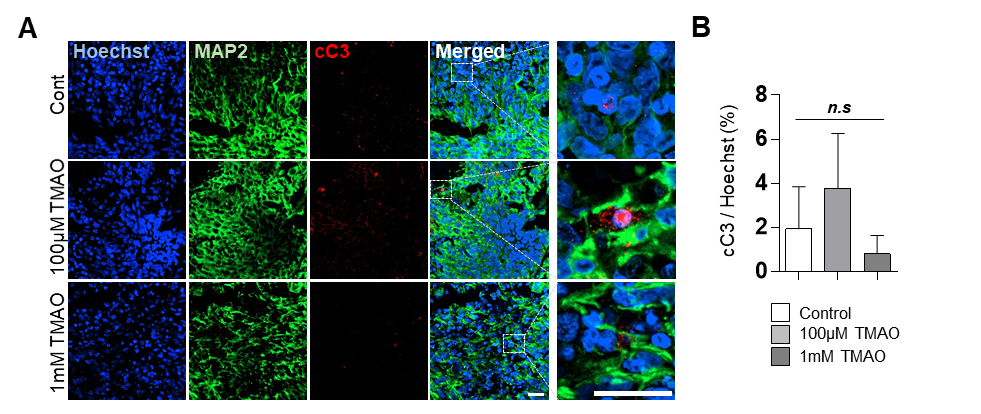


**FIGURE S1 | Apoptotic cell analysis in TMAO-treated midbrain organoids** (A) Representative IF images showing the MAP2 and cleaved caspase3 (cC3) in midbrain organoids at 30WM. (B) Quantification of cC3 normalized with Hoechst. Data are AVE±SD. (Scale bar = 20 μm)

**
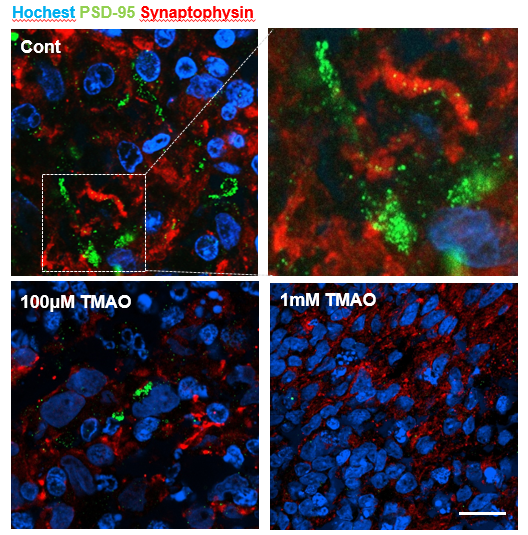
**

**FIGURE S2 | Synaptic marker analysis in TMAO-treated midbrain organoids.** Representative high-resolution images showing the PSD-95 and Synaptophysin in midbrain organoids at 8WM (Scale bar = 20 μm)


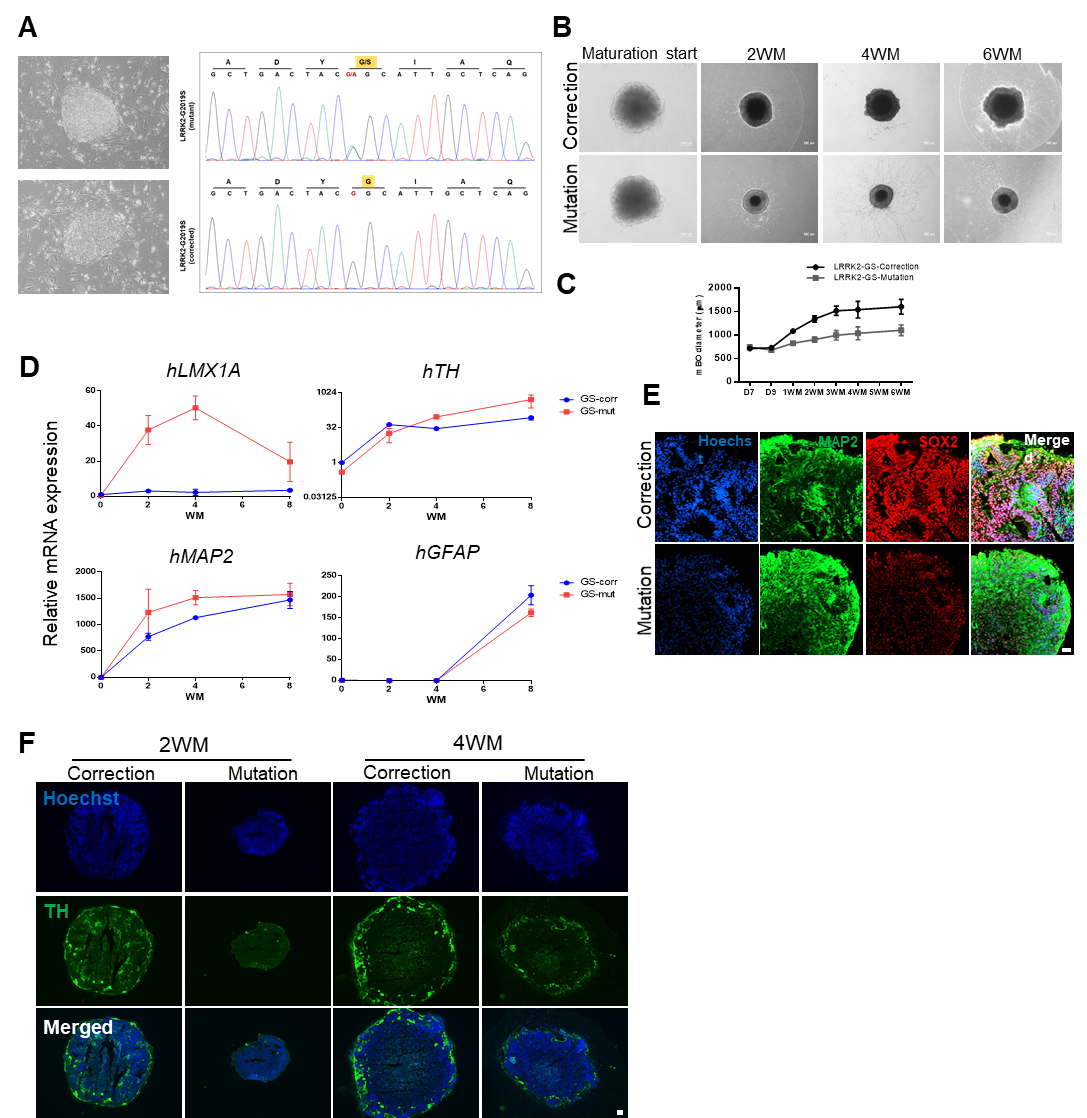


**FIGURE S3 | Generation of midbrain organoids from LRRK2-G2019S mutation and correction iPSCs for Parkinson’s disease modeling.** (A) gDNA sequencing results of LRRK2-G2019S mutation and correction iPSCs. (B, C) Phased contrast images of organoids size and quantification. (D) Relative mRNA expression of *SOX2, PAX3, LMX1a, TH, MAP2, GFAP* at day0, 2WM, 4WM, and 8WM of midbrain organoids. (E) Representative immunostaining images of MAP2 and SOX2 at 2WM/ (F) Representative immunostaining images of TH at 2WM and 4WM of organoids. (Scale bar = 20μm)


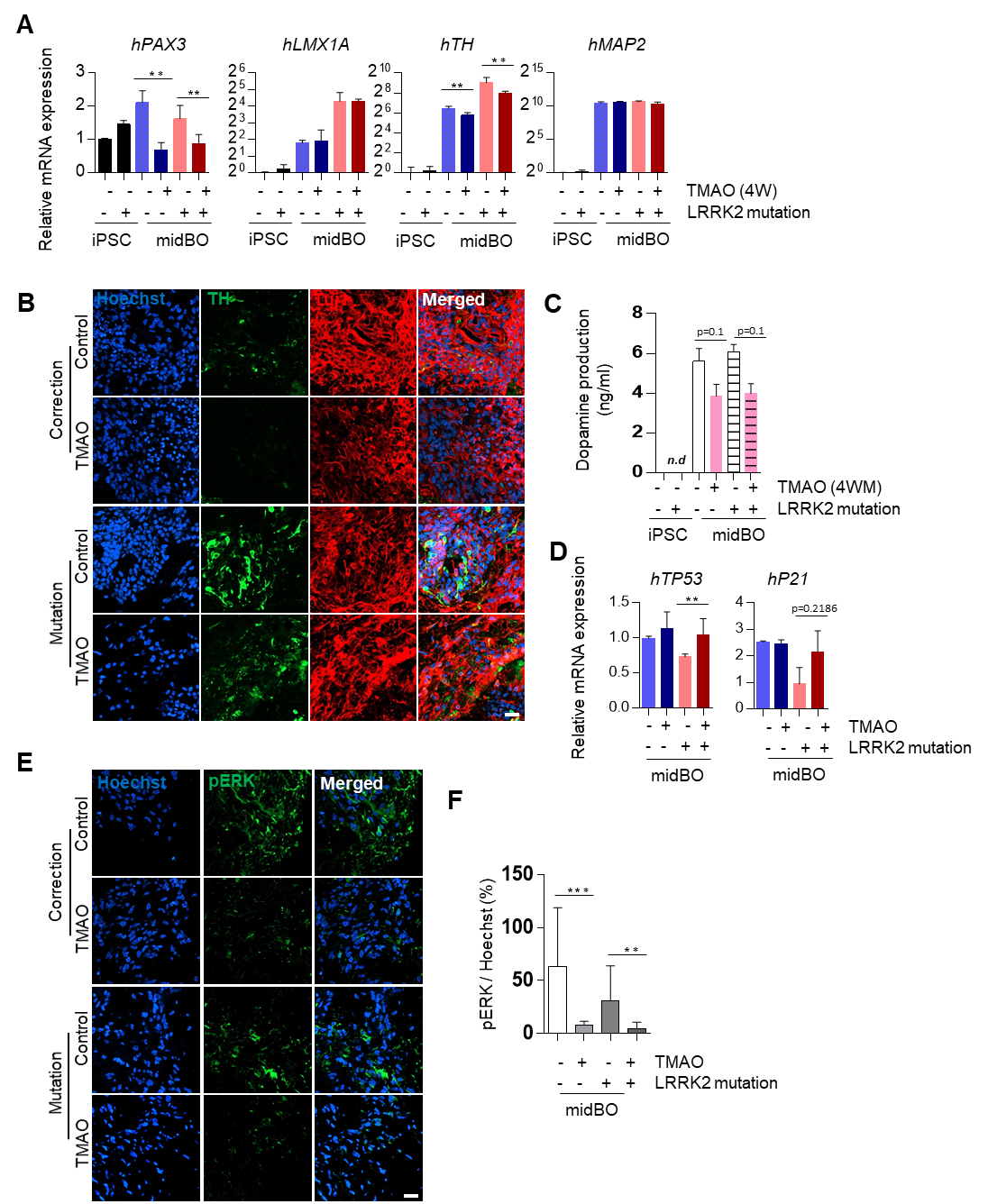


**FIGURE S4 | Aging and neurodegeneration associated phenotypes of TMAO-treated midbrain organoids.** (A) Relative mRNA expression levels of *PAX3, LMX1a, TH, and MAP2* at 4 weeks after TMAO treatment. (B) Representative immunostaining images of TH and TUJ1 at 8WM of midbrain organoids. (C) Quantification of dopamine secretion from iPSCs and midbrain organoids at 8WM. (D) Relative mRNA expression of *TP53* and *BAX*. (E, F) Representative immunostaining images of pERK and quantification data.
